# An Interaction between Glutathione and the Capsid Is Required for the Morphogenesis of C-Cluster Enteroviruses

**DOI:** 10.1371/journal.ppat.1004052

**Published:** 2014-04-10

**Authors:** Hsin-Chieh Ma, Ying Liu, Chunling Wang, Michael Strauss, Nina Rehage, Ying-Han Chen, Nihal Altan-Bonnet, James Hogle, Eckard Wimmer, Steffen Mueller, Aniko V. Paul, Ping Jiang

**Affiliations:** 1 Department of Molecular Genetics and Microbiology, Stony Brook University, Stony Brook, New York, United States of America; 2 Division of Infectious Diseases and Vaccinology, School of Public Health, University of California, Berkeley, Berkeley, California, United States of America; 3 Harvard Medical School, Boston, Massachusetts, United States of America; 4 Helmholtz Centrum München, Munich, Germany; 5 Department of Biological Sciences, Rutgers University, Newark, New Jersey, United States of America; Purdue University, United States of America

## Abstract

Glutathione (GSH) is the most abundant cellular thiol playing an essential role in preserving a reduced cellular environment. Cellular GSH levels can be efficiently reduced by the GSH biosynthesis inhibitor, L-buthionine sulfoximine (BSO). The aim of our study was to determine the role of GSH in the growth of two C-cluster enteroviruses, poliovirus type 1 (PV1) and coxsackievirus A20 (CAV20). Our results show that the growth of both PV1 and CAV20 is strongly inhibited by BSO and can be partially reversed by the addition of GSH. BSO has no effect on viral protein synthesis or RNA replication but it strikingly reduces the accumulation of 14S pentamers in infected cells. GSH-pull down assays show that GSH directly interacts with capsid precursors and mature virus made in the absence of BSO whereas capsid precursors produced under GSH-depletion do not bind to GSH. In particular, the loss of binding of GSH may debilitate the stability of 14S pentamers, resulting in their failure to assemble into mature virus. Immunofluorescence cell imaging demonstrated that GSH-depletion did not affect the localization of viral capsid proteins to the replication complex. PV1 BSO resistant (BSOr) mutants evolved readily during passaging of the virus in the presence of BSO. Structural analyses revealed that the BSOr mutations, mapping to VP1 and VP3 capsid proteins, are primarily located at protomer/protomer interfaces. BSOr mutations might, in place of GSH, aid the stability of 14S particles that is required for virion maturation. Our observation that BSOr mutants are more heat resistant and need less GSH than wt virus to be protected from heat inactivation suggests that they possess a more stable capsid. We propose that the role of GSH during enterovirus morphogenesis is to stabilize capsid structures by direct interaction with capsid proteins both during and after the formation of mature virus particles.

## Author Summary

Enteroviruses are plus stranded RNA viruses in the *Picornaviridae* family that cause as many as 3 billion infections per year. Enterovirus morphogenesis, which involves the encapsidation of newly made viral RNAs, has been studied for many years but the process is still poorly understood. Elucidation of this process is important for the development of drug treatments for a variety of human diseases. We describe the role of glutathione, an important cellular reducing agent, in enterovirus morphogenesis by studying the inhibition of GSH biosynthesis with BSO on viral proliferation. We discovered that GSH directly interacts with viral capsid precursors and the mature virus. In the presence of BSO the accumulation of a small capsid precusor (pentamer) is reduced and consequently no mature viruses are produced in virus-infected cells. Drug resistant viruses are easily isolated with mutations located in two of the capsid proteins, VP1 and VP3. We propose a model to explain the role of GSH in enterovirus morphogenesis, which is to stabilize the capsid precursors and the mature virus during and after the encapsidation of the progeny viral RNA by direct interaction with capsid proteins.

## Introduction

Glutathione (GSH), γ-L-glutamyl-L-cysteinylglycine, is an important cellular reducing agent, which prevents damage to cellular components caused by free radicals or peroxides. In addition, GSH has roles in signal transduction, gene expression and apoptosis [1]. The thiol group (SH) of GSH's cysteine serves as a proton donor and is responsible for the biological activity of GSH. Glutathione is present in several forms in cells, tissues, and plasma, at a high concentration of about 5 mM. It primarily exists in free form either in a reduced (GSH) or in an oxidized state (GSSG). GSH is also capable of forming disulfide bonds with cysteine residues in proteins and in its bound form it regulates protein function [1].

The cellular synthesis of GSH takes place in two consecutive steps. The first and rate-limiting step is the synthesis of a dipeptide, catalyzed by γ-glutamylcysteine synthase. In the second step glycine is added to the dipeptide in a reaction catalyzed by glutathione synthase. L-buthionine sulfoximine (BSO) is a specific and selective inhibitor of γ-glutamylcysteine synthase and consequently of GSH synthesis [2]. Pretreatment of HeLa cell monolayers with BSO reduces the total GSH level to <1% of control values by 48 hr post treatment [3]. Previous studies with BSO have shown that GSH influences viral replication. Specifically, BSO pretreatment of cells enhanced the replication of HIV, influenza virus, HSV-1 and Sendai virus [4]–[7]. In contrast, recently it was shown that BSO inhibits replication of coxsackievirus B3(CVB3), a B-cluster enterovirus, by blocking virus morphogenesis [3]. Early studies with PV have shown that in the presence of 5–10 mM glutathione the eclipse period of poliovirus infection is blocked [8]. Eclipse refers to an early phase of the viral replication cycle during which the metabolic machinery of the host is reorganized for the subsequent production of progeny virions. Adsorption and penetration of the virus to the host cell were not affected but the entering particles were not uncoated. Studies on the effect of BSO on Echovirus 9 proliferation indicated that the drug inhibited apoptosis induced by this virus [9]. Finally, GSH was found to stabilize purified poliovirus (PV) particles to *in vitro* heat inactivation, similarly to the action of S7, methylthiopyrimidine [10] or arildone, an antiviral agent that reversibly blocks the uncoating of PV [11].

**Figure 1:**
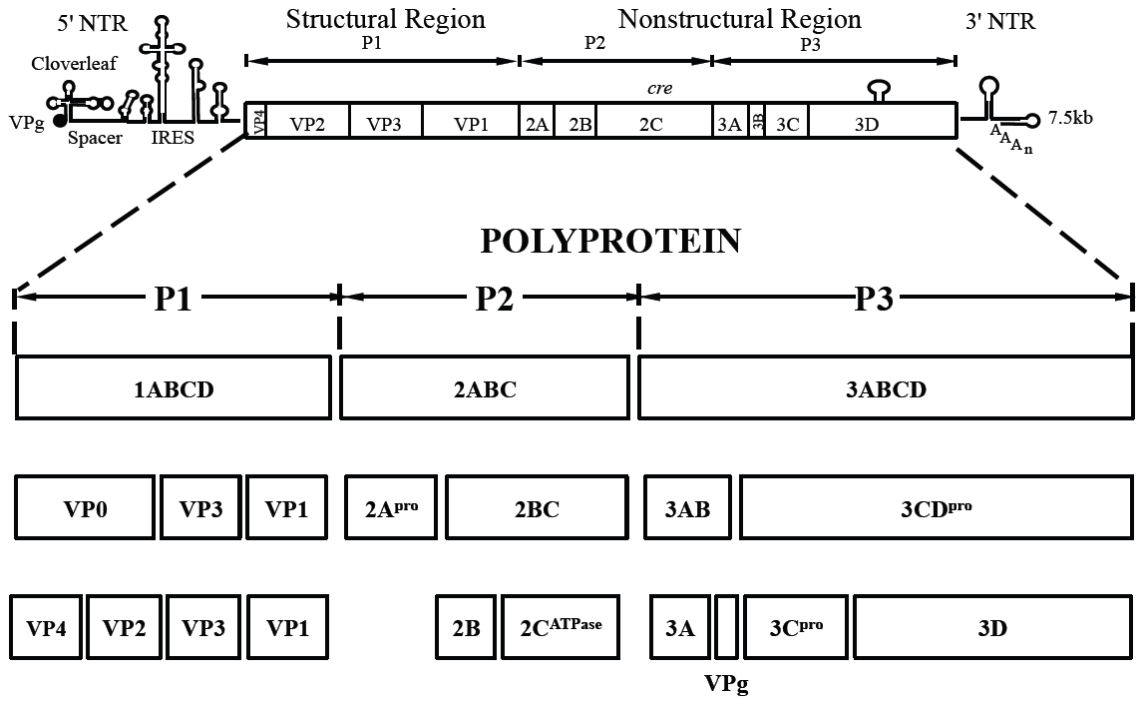
Genome structure of PV1 RNA and polyprotein processing. The PV1 genomic RNA contains a 5′NTR, a single open reading frame, a 3′NTR and poly (A) tail. The polyprotein, translated from a single open reading frame, has one structural (P1) and two nonstructural domains (P2, P3). The P1 domain is released from the polyprotein by 2A^pro^. Further processing of the P1 domain into VP0, VP3 and VP1 is by 3CD^pro^, followed by the maturation cleavage of VP0 into VP4 and VP2 by an unknown mechanism. The P2/P3 domains are processed by 3C^pro^/3CD^pro^ to generate different precursors and mature nonstructural proteins.

Poliovirus (PV) is a plus strand RNA virus in the *Enterovirus* genus of the *Picornaviridae*. The 7.5 kb genome of poliovirus contains a single open reading frame, which encodes one capsid (P1) and two nonstructural domains (P2 and P3) (Figure 1). The P1 precursor, after interacting with cellular chaperone Hsp90 [12] adopts a processing-competent conformation that is cleaved by viral proteinase 3CD^pro^ [13] into capsid proteins VP0, VP1 and VP3 (Table 1). These assemble to form a 5S protomer and then five protomers assemble yielding a 14S pentamer. Twelve pentamers make up the 75S empty viral capsid. The viral particle containing RNA, the provirion (150S), undergoes the final maturation cleavage of VP0 to yield VP2 and VP4. Whether the progeny RNA is inserted into the empty capsid or the pentamers surround and enclose the viral RNA remains an unanswered question. The experimental evidence available thus far favors the idea that 14S subunits are key assembly intermediates in PV1 morphogenesis. Studies using the *in vitro* cell-free translation/replication system demonstrated that 14S particles interact with newly made viral RNA and are assembled into virions [14]. In addition, electron microscopic analyses indicated that 14S particles associate with the replication complex and can be cross-linked to viral RNA by UV-irradiation [15]. Finally, Nugent and Kirkegaard observed that only 14S particles and mature virions interacted *in vitro* with poliovirus RNA but empty capsids did not [16]. The question of whether 75S empty capsids are the storage form of pentamers or dead-end products is still controversial [14], [17]–[20].

**Table 1:** Stepwise processing of the P1 capsid protein and maturation of the PV particle.

	P1	precursor protein
I	P1* (by associating with Hsp90)	processing-competent conformation
II	VP0, VP3, VP1	protomer 5S
III	(VP0, VP3, VP1)_5_	pentamer 14S
IV	[(VP0, VP3, VP1)_5_]_12_	empty capsid (procapsid) 75S
V	[(VP0, VP3, VP1)_5_]_12_RNA	provirion 150S
VI	[(VP4, VP2, VP3, VP1)_5_]_12_RNA	virion 150S

Although the basic steps involved in the encapsidation of poliovirus RNA are well established, very little is known about details of the overall process. We have recently made the interesting observation that the specificity of enterovirus encapsidation (PV, C-CAV) is determined by an interaction between capsid proteins and nonstructural protein 2C^ATPase^ [21]. An alanine scanning mutagenesis analysis of the 2C^ATPase^ polypeptide strongly supported this model and identified, in addition, a domain near the C-terminus that is required for morphogenesis. Indeed, mutations in VP1 and VP3 were found to compensate for the defect in encapsidation [22]. Our recent work led us to conclude that no RNA encapsidation signal is involved in poliovirus assembly [21], [22].

As part of our ongoing studies on the morphogenesis of C-cluster enteroviruses, we have examined the effect of GSH on encapsidation by using drug inhibition and biochemical experiments. In this study we observed that BSO blocked the production of both PV1 and CAV20 in tissue culture cells by more than hundred fold. Neither protein synthesis nor RNA replication were affected by the drug treatment. However, we observed that the level of 14S pentamers was severely reduced in the absence of GSH due to BSO-treatment. Consequently very little, if any, infectious mature virus was produced. Immunofluorescence cell imaging confirmed the absence of mature virus in drug-treated PV1-infected lysates even though capsid proteins colocalized with nonstructural proteins in the replication complex. GSH-pull down assays indicated that GSH physically interacts with the mature virus and capsid precursors. Drug resistant mutations map to capsid proteins VP1 and VP3. Structural analyses of capsids of BSO-resistant mutants indicate that the mutations are located at protomer/protomer interfaces of pentamers supporting a role for GSH in the stabilization of these precursors. Heat-inactivation studies confirm the proposed stabilizing effect of GSH on purified PV1 particles. Our results suggest that the effect of GSH on PV morphogenesis is specific and that the primary function of GSH is to stabilize the capsid proteins both during and after their assembly into the viral particle.

## Results

### BSO inhibits the growth of both PV1 and CAV20 on HeLa cells

**Figure 2:**
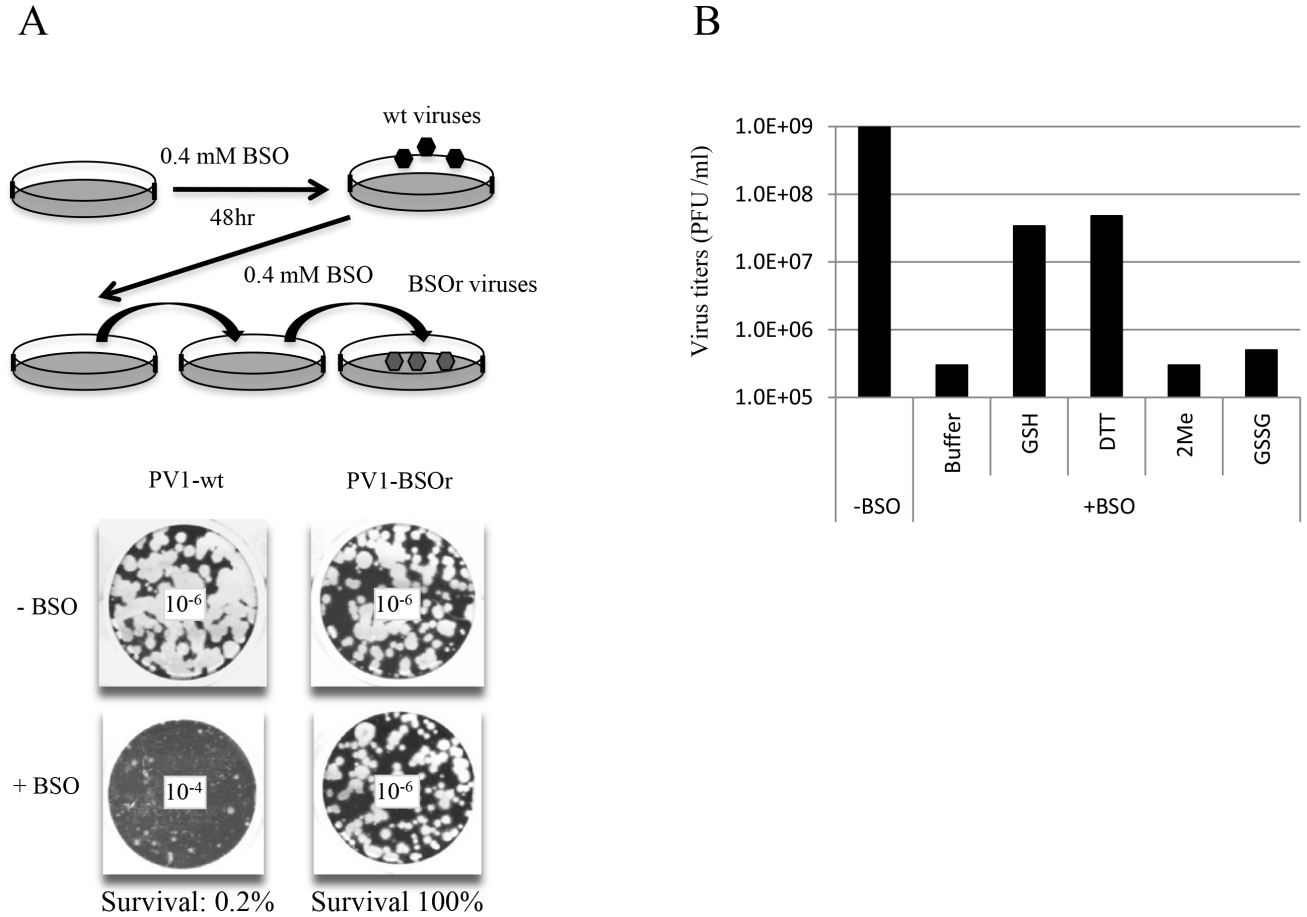
BSO-treatment of HeLa cells inhibits the growth of PV1. (**A**) HeLa cells were pretreated with 0.4 mM BSO for 48 hr and were infected with PV1-wt at a moi of 1. The cell lysates were passaged in the presence of the drug until BSOr mutants developed and CPE (cytopathic effect) was observed. BSOr and wt lysates were used to infect either untreated or drug-treated HeLa cells at a moi of 1 and incubation continued until CPE. Viral titers were determined by plaque assay, as described in Materials and Methods. Dilution factors used for the plaque assay are given in the center of the wells. (**B**) Reversal of BSO-inhibition of PV1 growth by GSH and DTT. BSO-treated HeLa cells were infected with PV1-wt at a moi of 1 in the presence of 5 mM different reducing agents (GSH, DTT and β-Me) or 5 mM GSSG. Cell lysates harvested at 8 hr post-infection were used to determine the virus titers by plaque assays (Materials and Methods).

In view of the relatively high cellular concentration of GSH (5 mM), HeLa cells have to be pretreated with BSO (0.4 mM) for 48 hr to reduce its concentration to below detectable levels [3]. We used these conditions to test the effect of BSO-pretreatment on poliovirus infection. In preliminary experiments untreated or BSO-pretreated HeLa cell cultures were infected with wild-type type 1 PV (PV1-wt) and incubated for 8 hr in the absence or presence of the drug, respectively. A measurement of the virus titer by plaque assays indicated a 100-fold reduction in the yield of virus produced in the presence of the drug (Figure 2A). In a parallel experiment a mixture of BSO resistant (BSOr) variants, isolated by passaging PV1-wt on HeLa cells in the presence of BSO, were also tested. The drug had no effect on the growth of the BSOr variants (Figure 2A).

Similarly, proliferation of CAV20-wt, another member of the C-cluster enteroviruses, was also found to be severely inhibited in BSO-treated HeLa cells (data not shown). BSO resistant CAV20 (CAV20-BSOr) mutants were also isolated on BSO-treated cells after several passages in the presence of 0.4 mM BSO although these mutants did not arise as easily as the PV1-BSOr mutants (data not shown). Together, these results suggest that GSH is required for the growth of both PV1 and CAV20 since BSO, the inhibitor of GSH synthesis, blocks virus production.

### Reversal of BSO-inhibition of PV1 growth *in vivo* by GSH

Next we tested whether the requirement for GSH during PV growth is specific or whether GSH can be replaced by other reducing agents, such as dithiothreitol (DTT), β-mercaptoethanol (β-ME) or GSSG, the oxidized form of GSH (Figure 2B). Untreated or BSO-pretreated HeLa cells were infected with PV1-wt and at 2 hr post infection various compounds (5 mM) were added to the cultures. Incubation continued for 8 hr in the absence or presence of BSO, respectively (Figure 2B). Both GSH and DTT but not GSSG, were able to reverse the inhibitory effect of BSO, albeit only partially. Partial rescue by DTT, however, does not necessarily mean that it can directly replace GSH-function in poliovirus growth and morphogenesis. DTT, a reducing agent frequently used in biochemical experiments, might function indirectly, for example, by reducing GSSG (that by itself does not rescue inhibition) to GSH. Surprisingly, β-ME, another common reducing agent in the laboratory, lacked the ability to overcome the inhibitory effect of BSO. It should be noted that in studies with CVB3 no reversal of the BSO-induced inhibition of viral growth was achieved by either DTT or by β-ME [3].

### BSO-resistant mutations are mapped to capsid proteins VP1 and VP3

**Figure 3:**
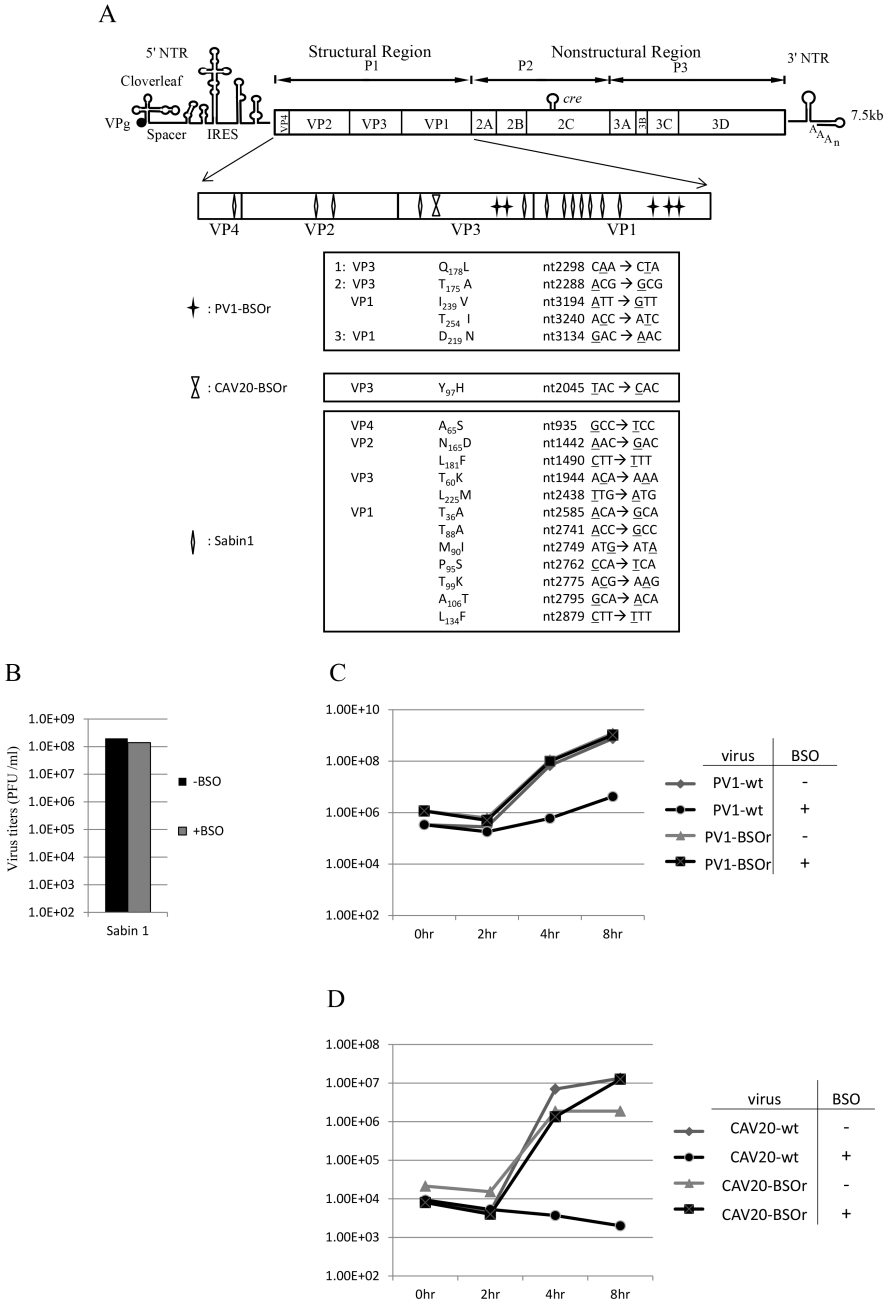
BSO resistant PV1 and CAV20 variants and their growth curves in HeLa cells. (**A**) Identification of BSO resistant mutations in the capsid of PV1 and CAV20. BSOr mutants of PV1 and CAV20 were plaque purified and after RT/PCR of the viral RNAs the cDNAs were sequenced in the capsid domain (Materials and Methods). The loci of the mutations in the capsid domain (enlarged) are illustrated. Also included are mutations in the capsid of PV type 1 Sabin, also a BSOr mutant. The residue or residues responsible for the BSOr phenotype of PV type 1 Sabin has not yet been identified. (**B**) The growth of PV type 1 Sabin is not inhibited by BSO. PV type 1 Sabin was grown in the absence or presence of BSO and the virus titers at 8 hr post infection were determined by plaque assay (Materials and Methods). (**C**) Untreated and BSO-pretreated cells were infected with PV1-wt or PV1-BSOr (VP3 Q178L) at a moi of 10. Cultures of infected cells were harvested a 0 hr, 2 hr, 4 hr, and 8 hr post-infection. Virus titers were determined by plaque assays (Materials and Methods). (**D**) Untreated and BSO-pretreated cells were infected with CAV20-wt or CAV20-BSOr (VP3 Y97H) at a moi of 1. Cultures of infected cells were harvested at 0 hr, 2 hr, 4 hr, and 8 hr post-infection. Virus titers were determined by plaque assays (Materials and Methods).

To map the mutations in BSO resistant variants of both PV1 and CAV20, the plaque-purified viruses were subjected to full-length genome sequence analyses. Three PV1-BSOr variants revealed an array of mutations in capsid proteins VP1 and VP3, whereas only a single VP3 mutation was found in 4 different plaque-purified CAV20-BSOr mutants (Figure 3A).

H. A. Thibaut and his colleagues have recently observed that the small compound TP219 inhibits the replication of several enteroviruses by binding GSH (Thibaut et al. in press). Interestingly, the replication of the poliovirus vaccine strain Sabin 1 was resistant to TP219 (Thibaut et al, submitted for publication). Since TP219 depletes available cellular GSH we have followed Thibaut's observation and found that, indeed, PV type 1 Sabin virus is also resistant to depletion of GSH by BSO (Figure 3B). The Sabin 1 vaccine strain was initially isolated following numerous passages of its progenitor strain PV1(Mahoney) in different non-human tissue culture cells and monkeys [23]. The passages lead to 21 amino acid changes in the viral polyprotein, of which 12 are located in the capsid domain [24] (Figure 3A). Only 4 of the 12-capsid mutations have been assigned to the attenuated phenotype of the PV type 1 Sabin vaccine [23]. We do not know yet which of the 12-capsid mutations in PV type 1 Sabin are responsible for the BSOr phenotype.

### Growth curves of PV1 and CAV20 on HeLa cells in the presence of BSO

To examine the effect of BSO on the growth of C-cluster enteroviruses in more detail we analyzed growth curves of wt and of drug-resistant PV1 (VP3 Q178L) and of CAV20 (VP3 Y97H) on HeLa cells, which were untreated (−BSO) or treated with the drug (+BSO). HeLa cell cultures were infected at a moi of 10 with wt or drug resistant variants of PV1 and incubation was with or without BSO, as indicated. Virus titers were monitored by plaque assay at various times, up to 8 hr post-infection. As shown in Figure 3C, PV1-infected control cells yielded 10^9^ PFU/ml but the drug reduced the yield about 100 fold at 4 hr and 8 hr post infection. In contrast, the depletion of GSH did not affect the growth of PV1-BSOr variant (VP3 Q178L) (Figure 3C). Essentially the same results were obtained with CAV20, except that drug treatment reduced the titer of the wt virus nearly 10,000-fold (Figure 3D). Again, the growth of CAV20-BSOr variant (VP3 Y97H) was unaffected in GSH depleted cells, and in the presence of BSO, at any time post infection (Figure 3D). It should be noted that the extent of inhibition did not significantly vary with the multiplicity of PV1 infection (0.1–10) (data not shown). These results confirmed our preliminary finding (Figures 2A and 2B) that GSH is required for the growth of both PV1 and CAV20, two C-cluster enteroviruses. As shown on Figure 3C, the PV1 virus titer slowly increased from 4–8 hr post infection in drug-treated cells in the presence of the drug. In order to determine whether the virus obtained at 8 hr post infection (Figure 3C) in the presence of BSO were BSOr variants or just a few wt viruses that escaped the inhibition by the drug, we tested the growth properties of the progeny PV1 obtained at 8 hr post infection in both untreated and BSO-pretreated HeLa cells. We observed that >90% of total population consisted of BSOr variants (data not shown), an observation indicating that drug resistant PV1 variants appear readily when GSH is depleted in the presence of BSO. In contrast, the virus titer for CAV20-wt in the presence of BSO did not increase (Figure 3D). This is in agreement with the result in Figure 3A showing that PV1 can easily become resistant by acquiring different mutations whereas only one VP3 mutation has been found in CAV20 that confers it the resistance to BSO. As mentioned previously, this suggests that CAV20 is much more sensitive than PV1 to suboptimal levels of GSH such that selection to resistance is more difficult.

### Structural analyses of BSOr mutations within the PV1 capsid

**Figure 4:**
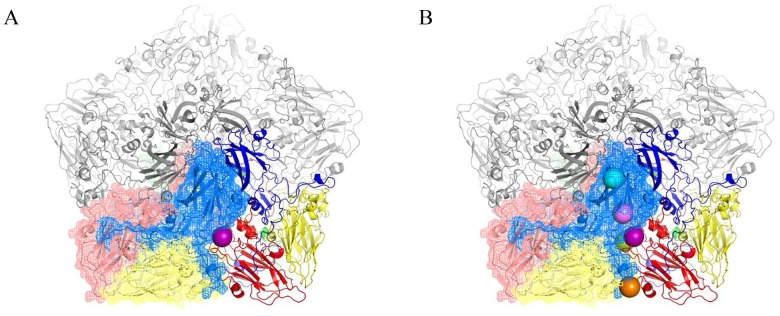
BSOr mutations are located at the interface between protomers. The poliovirus protomer, shown here as a colored ribbon with VP1 in blue, VP2 in yellow and VP3 in red, forms an interface with symmetry related neighbors, one of which is shown as a mesh with the same color scheme. Three remaining symmetry copies are shown in grey to make up the pentameric arrangement around the 5-fold axis. The locations of the mutations are shown as colored spheres and are predominantly found at the interface between protomers. (A) VP3 Q178L mutation alone; (B) All BSOr PV mutations. Colors: VP3 Q178L-purple; VP1 D219N-Orange; VP3 T175A-Green; VP1 I239V-Pink; VP1 254I-Cyan. Molecular graphics and analyses were performed with the UCSF Chimera package and PyMOL Molecular Graphics System, Version 1.5.0.4 Schrodinger, LLC. Chimera is developed by the Resource for Biocomputing, Visualization, and Informatics at the University of California, San Francisco (supported by NIGMS 9P41GM103311) [26], [43].

The existence of BSO resistant PV1 or CAV20 variants indicates that stable 14S particles and mature virus can be generated even in the absence of GSH. Therefore, it is reasonable to assume that the BSO resistant mutations have a role in stabilizing the mature virus and likely also one or more of the capsid precursors. Therefore we determined the exact location of the Q178L mutation mutations (Figure 4A) and also of all the other PV mutations: T254I/VP1, I239V/VP1, T175A/VP3) (Figure 4B) within the known atomic structure of the capsid [25]. Figure 4A illustrates the PV protomer, shown here as a colored ribbon, which forms an interface with symmetry related neighbors. The location of the Q178L mutation is shown with two nearby protomers highlighted. The other mutations are shown in Figure 4B, with two protomers highlighted. These mutations map primarily at protomer/protomer interfaces within the pentamers, an observation supporting our hypothesis that they contribute to the formation of a stable capsid even in the absence of adequate concentrations of GSH. Similarly, the CAV20 Y97H mutation is predicted to be at or near the Q178L mutation in the structure of a modeled CAV20 capsid (data not shown). The Q178L and Y97H mutations are located in the GH loop of VP3 both in the PV structure and the predicted CAV20 structure. Interestingly, the GH loop undergoes major structural rearrangements in the 135S cell entry intermediate of PV suggesting the possibility that the mutations also play a role during expansion or uncoating [26].

### BSO does not affect CAV20 protein translation or viral RNA synthesis

**Figure 5:**
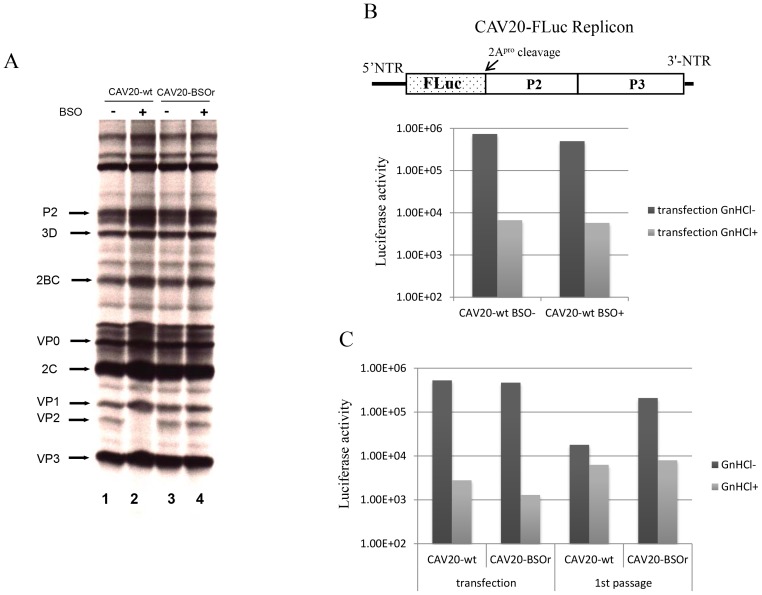
The effect of BSO on CAV20 protein translation, RNA replication and transencapsidation. (**A**) *In vivo* ^35^S labeling of CAV20-wt and CAV20-BSOr viral proteins in the absence and presence of BSO in HeLa H1 cells. Viral proteins were separated on SDS-polyacrylamide gels (12.5%), as described in Materials and Methods. (**B**) The genome structure of the CAV20 FLuc replicon used in the experiment is shown above. The FLuc coding sequence was used to replace the P1 domain of the polyprotein. RNA transcripts of the replicon were transfected into untreated or BSO-treated HeLa H1 cells either in the absence or presence of GnHCl (2 mM). Luciferase activity was measured at 9 hr post transfection. (**C**) CAV20 FLuc replicon RNA transcript was transfected into BSO-treated HeLa H1 cells either in the absence or presence of GnHCl. At 1 hr post-transfection the cells were superinfected either with wt or with CAV20-BSOr (VP3 Y97H) at a moi of 0.5. The cells were lysed at 6 hr post-infection and were then used to re-infect fresh HeLa H1 cells in the absence or presence of GnHCl.

To determine the stage of the viral life cycle at which adequate GSH concentrations are required for virus proliferation, we labeled the viral proteins with S^35^ trans-label in CAV20-wt infected HeLa cells that were untreated or pretreated with BSO, and analyzed ^35^S trans-labeled viral proteins by SDS-PAGE. BSO had no effect on the translation and processing of the CAV20 polyprotein. Similar to previously reported results [3] no maturation cleavage of CAV20 VP0 to VP2 and VP4 could be observed in cells depleted of GSH (Figure 5A, compare lanes 1 and 2), an observation indicating that no infectious particles were formed. In contrast the CAV20-BSOr variant produced similar levels of VP2 in the absence or presence of the drug (Figure 5A, compare lanes 3 and 4). We conclude that BSO treatment of HeLa cells has no apparent effect on CAV20 protein synthesis and processing.

The effect of GSH-depletion on viral RNA synthesis was examined with a CAV20-FLuc replicon in which the capsid domain of CAV20 was replaced with the FLuc coding sequence (Figure 5B). BSO-treated or untreated cells were transfected with RNA transcripts of the CAV20-FLuc replicon both in the absence or presence of guanidine hydrochloride (GnHCl), a potent inhibitor of RNA replication [27]. The luciferase signal in the presence of GnHCl, measures the level of translation of the transfected replicon, which in BSO treated and untreated cells, was at normal levels. RNA replication, on the other hand, was evaluated by measuring luciferase activity in the absence of GnHCl. Since the luciferase activity, in the absence of GnHCl, was comparable in BSO-treated and untreated cell cultures (Figure 5B) we conclude that GSH depletion and BSO have no inhibitory effect on RNA replication.

### BSO inhibits the morphogenesis of CAV20

To test the effect of GSH-depletion on encapsidation we performed trans-encapsidation assays [17]. Specifically, we measured the ability of BSOr viruses to trans-encapsidate a CAV20 FLuc replicon. Only those replicons that have been encapsidated during the transfection step by the capsid of the superinfecting BSOr virus are expected to produce a Luc signal during passage on fresh HeLa cells. HeLa cells were treated with BSO before transfection with transcript RNAs of a CAV20 FLuc replicon (Figure 5C) and incubated in the presence of the drug. Six hours post transfection the cells were superinfected with either CAV20-wt or CAV20-BSOr virus, both in the absence and presence of GnHCl. After 6 hr the cells were lysed and an aliquot was used to measure luciferase activity. Luciferase activity from transfection (-GnHCl), which measures RNA replication, was comparable in the cell lysates co-infected with CAV20-wt or CAV20-BSOr viruses (Figure 5C), confirming our preliminary finding that GSH-depletion has no inhibitory effect on RNA replication (Figure 5B). To measure encapsidation, an aliquot of the cell lysate was used to infect fresh HeLa cells and, again, luciferase activity was measured at 6 hr post infection. Those replicon-transfected cells that were superinfected with wt virus produced only background levels of Luc signal indicating a lack of trans-encapsidation by the wt capsid. In contrast, high luciferase activity was detected when superinfection was done with the BSOr virus, indicating that the capsid derived from the BSOr virus was able to transencapsidate the replicon. These results indicate that BSO-induced inhibition of viral growth functions at the level of morphogenesis.

### Binding between GSH and capsid proteins is essential for virus production

**Figure 6:**
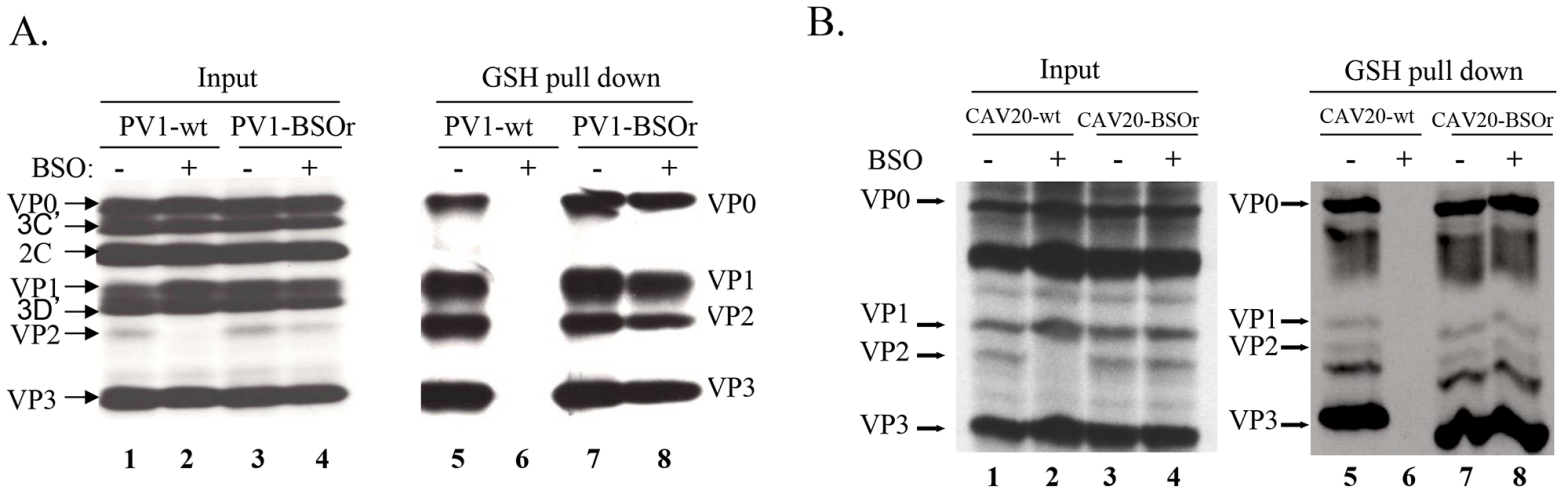
GSH directly interacts with capsid proteins of both PV1 and CAV20. (A) GSH-pull down assay of PV1-infected lysates. HeLa cells, untreated or BSO-treated, were infected with PV1-wt or PV1-BSOr (VP3 Q178L). (B) GSH-pull down assay of CAV20-infected lysates. HeLa cells, untreated or BSO-treated, were infected with CAV20-wt or CAV20-BSOr. The viral proteins were labeled with ^35^S-Translabel from 4 to 5 hr post-infection and the cells were harvested. The viral proteins in an aliquot of the lysates were analysed on SDS-polyacrylamide (11.5%) gels (lane 1–4). Samples were loaded onto GSH Sepharose beads and the pulled down material was analyzed by SDS-PAGE (lane 5–8).

The presence of mutations in VP3 and VP1 in the BSOr variants (Figure 3A) suggested the possibility that GSH directly interacts with the wt capsid precursors or virus particles in infected cells. To examine this possibility, we did GSH-pull down assays using ^35^S-labeled lysates of untreated or BSO-treated HeLa cells that have been infected with wt or BSOr PV1 and CAV20 viruses (Figure 6A and 6B). The left panel of Figure 6A shows labeled PV1 viral proteins of the input lysates separated by SDS-PAGE (Figure 6A, lanes 1–4). When the lysates were applied to GSH-Sepharose beads and the bound material was analyzed again by SDS-PAGE (Figure 6A, right panel), the pull down mixtures from BSO-untreated cells infected with PV1-wt or PV1-BSOr were resolved into VP0, VP1, VP2 and VP3 (Figure 6A lanes 5 and 7). VP4, the smallest capsid protein, would not be visible under the conditions of the gel. The observation that both VP2 and VP0 were present in the mixture indicated that both mature virus and one or more capsid intermediate interacted with GSH. In BSO-treated cells, VP2 was not detectable in the lysates of cells made from infection with the PV1-wt virus (Figure 6A, left panel lane 2). This indicates that very little, if any, mature virus was formed under these experimental conditions because no VP0 was cleaved to VP2 and VP4. Moreover, no capsid proteins were present in the pull down of cell lysates from BSO-treated cells infected with PV1-wt virus (Figure 6A, lane 6). These results suggested either that no normal capsid intermediates were made or that these structures lost their ability to bind to GSH. In the presence of BSO, the PV1-BSOr variant yielded all viral capsid proteins in the GSH-pull down (Figure 6A, lane 8). This result suggests that capsid proteins of the BSOr variants, regardless of the absence of adequate amount of GSH, possess the correct conformation for the binding of GSH. Meanwhile, we observed that the viral proteins of CAV20-wt and CAV20-BSOr virus (Figure 6B, lanes 1–4) share a similar binding pattern to GSH (Figure 6B, lanes 5–8) as their PV1 counterparts, indicating that the interaction of GSH to capsid proteins is a general phenomena within C-cluster enteroviruses. The fact that the loss of binding of capsid proteins to GSH in PV1-wt leads to its failure to generate viable virus strongly suggests that the interaction of capsid proteins with GSH is important for virus production.

### BSO treatment of HeLa cells reduces the level of 14S pentamers and alters the mobility and stability of 150S particles

**Figure 7:**
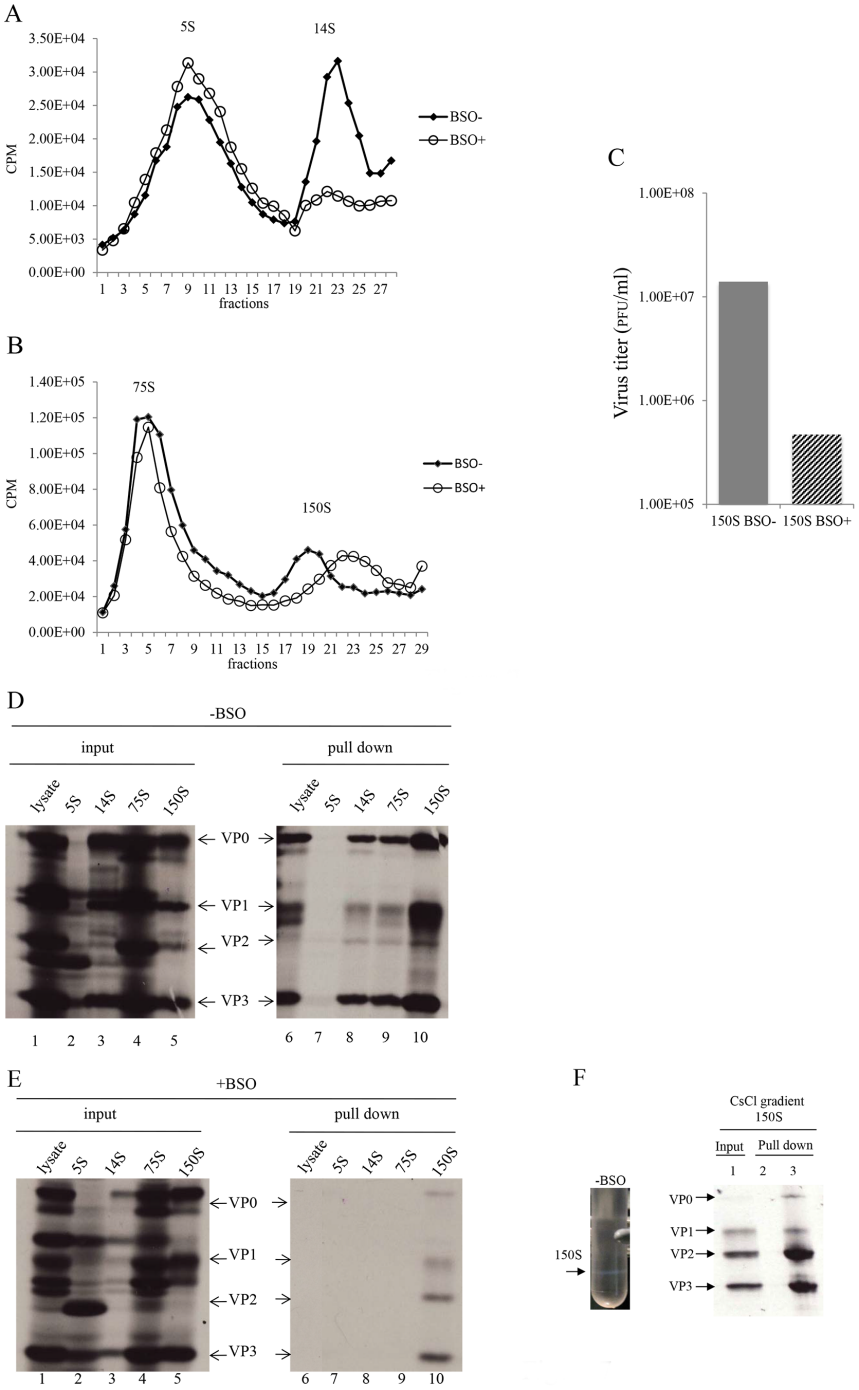
GSH interacts with encapsidation intermediates and mature virus. (**A**) Separation of 5S and 14S capsid intermediates on a sucrose gradient. Untreated and BSO-treated HeLa cells were infected with PV1-wt (moi 10) and metabolically labeled with ^35^S-Translabel from 4–6 hr post-infection. Cell lysates were made and were analyzed on a 6–25% sucrose gradient for the separation of 5S and 14S intermediates (Materials and Methods). Fractions were collected from the top and the radioactivity in each fraction was determined by scintillation counting. (**B**) Separation of 75S and 150S particles on a sucrose gradient. Untreated and BSO-treated HeLa cells, infected with PV1-wt (moi 10) were labeled with ^35^S-Translabel, as described above, and the cell lysates were analyzed on a 15–30% sucrose gradient for the separation of the 75S and 150S intermediates (Materials and Methods). Fractions were collected from the top and counted by scintillation counting. (**C**) Comparison of virus titers of 150S peak fractions derived from −BSO and +BSO gradients. 10 µl of 150S fractions, derived from both BSO-treated and –untreated infections, were titered by plaque assay (Materials and Methods). (**D**) GSH-pull down assays of capsid intermediates and of virus derived from −BSO lysates. Three fractions of each of the 5S, 14S, 75S and 150S peaks were pooled and an aliquot of each was analyzed by SDS-polyacrylamide (11.5%) gel electrophoresis (lanes 1–5). Only one part of the gel is shown, which contains the major capsid proteins. Lanes 6–10 show the proteins pulled down by GSH beads from the lysates and from the intermediate peaks. (**E**) Lanes 1–5 show the input ^35^S-labeled viral proteins in +BSO lysates and its capsid intermediates. Lanes 6–10 show proteins pulled down from the lysates or from the capsid intermediate peaks by GSH-Sepharose (Materials and Methods). (**F**) ^35^S-labeled lysates made in the absence of BSO were purified and subjected to CsCl centrifugation (Material & Methods). The virus band (−BSO) containing mature virions was isolated and an aliquot was applied to GSH Sepharose beads. The pull down sample was analyzed by SDS PAGE. Sepharose 4B beads were used as control.

With the aim of determining which step of particle assembly is inhibited by BSO we applied ^35^S-labelled PV1-wt infected HeLa cell lysates, made in BSO treated or untreated cells, to sucrose gradients to separate the 5S, 14S, 75S and 150S fractions (Figures 7A and 7B). It should be noted that the 150S fraction usually contains both provirions and mature virions. Lysates prepared from infected but BSO-untreated HeLa cells yielded gradients with distinct peaks of all intermediate structures and virus particles [28]. In contrast, the gradients made with lysates of BSO-treated cells exhibited normal 5S and 75S peaks but strikingly lower amounts of the 14S peak (Figure 7A). There are several possible explanations for the reduced amounts of 14S material visible on the gradient. First, there might be fewer pentamers made under conditions of insufficient GSH. In this case the production of subsequent capsid precursors, 75S and 150S, would have been decreased accordingly. However, the sucrose gradient profiles clearly showed that the peaks of 75S and 150S particles, in the presence of BSO, are comparable to their counterparts generated in the absence of the drug. Alternatively, the 14S pentamer particles are produced in normal amounts but are unstable in the absence of sufficient amounts of GSH. This explanation supports our hypothesis that the role of GSH during morphogenesis is to stabilize the 14S pentamers but it does not explain the seemingly normal production of 75S particles. As will be seen below in the GSH-pull down assay (Figure 7E), the properties of 75S particles produced in GSH-depleted HeLa cells are indeed different from those made in untreated HeLa cells.

We observed reproducibly in experiments with GSH-depleted cells a 150S peak, which migrated faster than the 150S peak obtained in untreated lysates (Figure 7B). As far as the shift in mobility of the 150S particle is concerned, these results suggest a malformation of the 150S particle, likely producing noninfectious virus particles. To test this hypothesis, we compared the virus titers in the 150S peaks, derived from lysates made in untreated or BSO-treated HeLa cells. Indeed, the infectious virus titer in the +BSO 150S peak was only about 3% of the plaque forming units generated under normal growth conditions (−BSO) (Figure 7C). The combined results of the sucrose gradients, especially the abnormal profiles of the 14S and 150S particles, suggest that 14S pentamers formed under GSH-depletion might have a different conformation that renders them unstable and unable to form normal 150S, even though they are still able to associate with the viral RNA.

### GSH interacts with capsid precursors

In Figure 6A we showed that VP0 was present in the GSH-pull down mixture indicating that one or more capsid intermediates interacted with GSH. To determine which capsid precursor binds to GSH we individually pooled three fractions of each peak, containing the highest levels of ^35^S label (Figures 7A and 7B), and subjected aliquots to GSH-pull down assays. The viral capsid proteins present before (input) and after pull down with GSH (pull down) were analyzed on SDS-PAGE (Figures 7D and 7E). Lysates made from untreated HeLa cells yielded the expected capsid proteins after GSH-pull down in all peaks except the 5S. This 5S peak contained primarily unidentified protein bands suggesting that most of the label in the peak (Figure 7A) is not capsid related (Figure 7E, lanes 2 and 7). After the pull down of lysates made in GSH-depleted cells no capsid proteins were detected from the 5S, 14S and 75S peaks and only trace amounts of capsid proteins were visible in the 150S peak (Figure 7E, lanes 7–10). A comparison of protein band intensities in the 150S peaks in lane 10 of Figure 7D, and Figure 7E suggests that these proteins are derived from the BSOr variants, which represent 3% of the normal virus titer as shown in Figure 7C. Overall these results suggest that GSH can bind to capsid intermediates under normal conditions. This binding might be crucial for morphogenesis because it confers the 14S pentamer a stable conformation that is required for forming normal 150S particles, which can mature into infectious virions.

### GSH binds to mature virus particles

The presence of VP2 in the GSH-pull down mixture suggested that GSH might also bind to the virion (Figure 6B). To confirm the interaction of GSH with mature virion particles, CsCl-purified mature viruses (Figure 7F, lane 1), made in the absence of BSO, were subjected to GSH-Sepharose pull down assays and the bound viral proteins were analyzed by SDS-PAGE (Figure 6F). As a control we also analyzed also empty Sepharose beads, which yielded no visible bands (Figure 7F, lane 2). The bound proteins in the purified virus preparation consisted of the expected amounts of VP1, VP2, and VP3 (Figure 7F, lane 3). It should be noted that the amount of labeled amino acids (Cys+Met) in VP1 (7) is about one half of what it is in VP2 (14) or VP3 (17), therefore the VP1 band in the gel appears significantly lighter than the other two bands. A trace of uncleaved VP0 in the GSH-pull down suggests the presence of a small amount of provirion in the virus band. These results confirm a direct binding between GSH and the mature virus and possibly also the provirion.

Lysates made from cells infected with PV1-wt, under GSH-depletion condition, did not yield any virus band in a parallel CsCl gradient (data not shown). The lack of a band at the expected location of the gradient indicates that the 150S particles made under these conditions are unstable and fall apart, probably during our purification procedure that includes a strong detergent, SDS.

### Immunofluorescence cell imaging of BSO-untreated or -treated HeLa cells infected with wt and BSOr viruses

**Figure 8:**
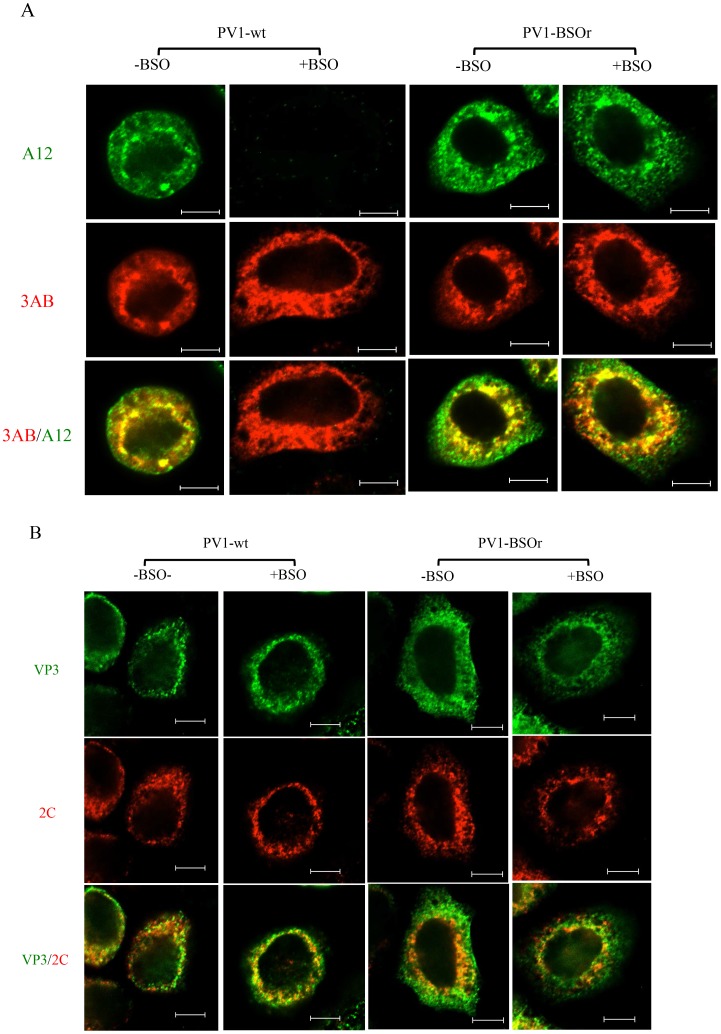
Immunofluorescence cell imaging of PV1-infected HeLa cells in the presence and absence of BSO. (**A**) HeLa cells were infected with PV1-wt or PV1-BSOr (VP3 Q178L) at a moi of 10 at 37°C. After 4 hours incubation, the infected cells were probed for mature virus with A12 primary antibody (anti-PV human serum), which binds specifically to mature virus and provirions (Nihal Altan Bonnet, unpublished results), and Alexfluor 488-conjugated secondary antibody (green color). The localization of 3AB, a member of the replication complex, was determined in the same cell using 3AB mouse monoclonal antibody and Alexfluor 555-conjugated secondary antibody (red color). The scale is 5 µm. (**B**) Infected cells were probed for capsid precursors using VP3 polyclonal antibody and Alexfluor 488-conjugated secondary antibody (green color). The localization of 2C^ATPase^, another nonstructural protein on the replication complex, was determined in the same cell using 2C mouse monoclonal antibody and Alexfluor 555-conjugated secondary antibody (red color).

To confirm that mature virus is not formed in BSO-treated infected cells, we used immunofluorescence cell imaging. Untreated or BSO-treated HeLa cells were infected with PV1-wt (moi 10). At 4 hr post infection the cells were probed for the presence of mature virus [with monoclonal antibody (mcAb) A12] and for 3AB (with mc3AB), the latter being an essential viral protein component of the replication complex. In untreated cells (Figure 8A, −BSO lane), mature virus and 3AB colocalize at the peri-nuclear region of the infected cell whereas no mature virus particles were detected under the conditions of the experiment in drug-treated cells (Figure 8A, +BSO lane). In a similar experiment with a PV1-BSOr variant (VP3, Q178L), mature viruses were observed in equal amounts in untreated or drug-treated cells (Figure 8A, −/+BSO lanes).

As we mentioned before, the 150S peaks derived from GSH-depleted lysates (+BSO) were present in about the same amount as the ones made in untreated cell lysates (Figure 7B). These results suggested that BSO has no direct effect on the interaction between capsid precursors and the protein components of the replication complex. To confirm this observation we again used immunofluorescence cell imaging to examine the localization of VP3 (with VP3 polyclonal antibody) and of 2C^ATPase^ (with 2CmcAb) in infected cells that were either untreated or BSO-treated. A specific VP3/2C^ATPase^ interaction has recently been reported by us to be essential for poliovirus morphogenesis [21], [22]. Our results clearly showed normal colocalization of VP3 and of 2C^ATPase^, both in BSO-treated and untreated cells (Figure 8B). The same results were obtained when PV1-BSOr (VP3, Q178L) was used instead of the wt virus (Figure 8B). The successful localization of the capsid precursors to the replication complex in BSO-treated cells suggest that the structural (conformational) change in the capsid precursor due to GSH-depletion does not affect the direct protein-protein interactions between the VP3-component of capsid precursors and 2C^ATPase^ on the replication complex.

### GSH protects purified poliovirus particles from heat inactivation *in vitro*

**Figure 9:**
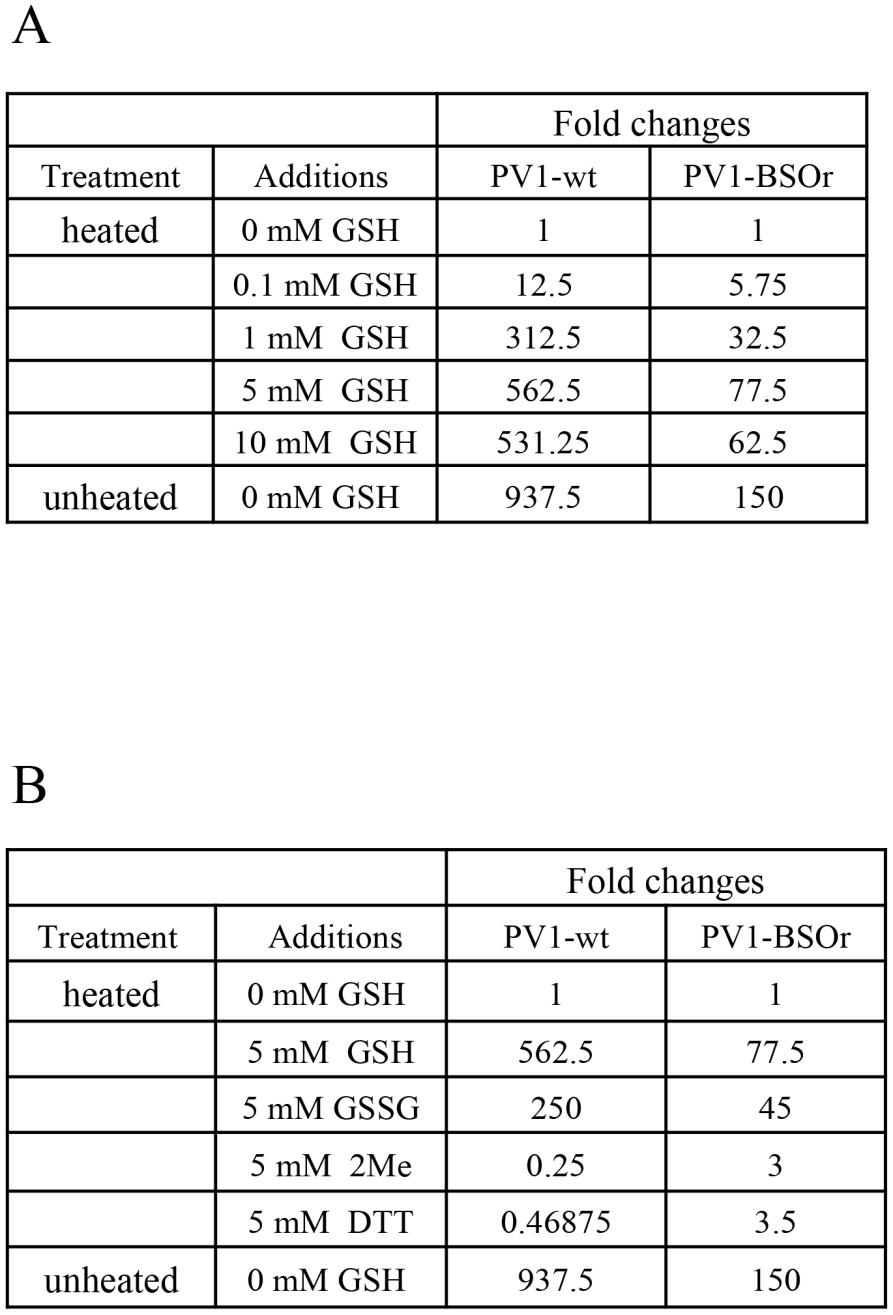
GSH protects PV1-wt and PV1-BSOr from heat inactivation. (**A**) Protection from heat inactivation of PV1-wt or PV1-BSOr by GSH. Purified PV1-wt or PV1-BSOr (VP3, Q178L) 3×10^9^ PFU was incubated *in vitro* in PBS either in the absence or presence of various amounts of GSH for 25 min at 48°C. The amount of virus remaining after the incubation was determined by plaque assays. The virus titers obtained after heating without GSH for both viruses were taken as “1”. (**B**) Protection from heat inactivation of PV1-wt or PV1-BSOr by reducing agents or GSSG. PV1-wt or PV1-BSOr 3×10^9^ PFU were incubated in PBS either in the absence or presence of various reducing agents (5 mM) or of GSSG (5 mM) (Materials and Methods). The virus titer obtained after heating without GSH for both viruses were taken as “1”.

Early studies with poliovirus have shown that methylthiopyrimidine, S7, and GSH can protect the virus from heat inactivation *in vitro* [10]. To examine the protective role of GSH during heat inactivation of PV1, we carried out a series of *in vitro* experiments with both PV1-wt and a PV1-BSOr variant (VP3 Q178L). The viruses, purified on sucrose cushions, were incubated at 48°C for 25 minutes, in the absence or presence of GSH, and the fold change in titer was examined (Figure 9A). For either PV1-wt or PV1-BSOr virus, the results are presented as “fold change” relative to the virus titer obtained after heat inactivation in the absence of GSH, which is taken as “1”. During heating the titer of the wt virus was reduced about 1000-fold while that of the BSOr changed only 150-fold. When GSH was present during heating, both wt and BSOr viruses were protected in a dose-dependent manner from 0.1 mM to 5 mM. GSH provided stronger protection to the wt virus than to the BSOr variant. GSSG, the oxidized form of GSH, was also able to protect the wt or BSOr viruses from heat-inactivation (Figure 9B) but neither β-ME nor DTT (5 mM) provided such protection (Figure 9B). These results suggest that protection from heat inactivation requires a direct interaction between GSH or GSSG and the virus particles but a reducing environment is not sufficient. In addition they suggest that the BSOr mutants are more resistant to heat inactivation than the wt virus and less dependent on GSH than the wt, likely because the BSOr capsids are more stable. In combination with the results in Figure 7F, we conclude that the function of the interaction between GSH and mature viral particles *in vitro* is to stabilize the virions.

## Discussion

An effect of intracellular GSH concentration on viral proliferation is known for several years [3]–[7], [29] but the mechanism of GSH's influence on viral metabolism is largely obscure. In most cases reducing intracellular GSH concentration aids viral proliferation and pathogenesis but Smith and Dawson (2006) reported a strong inhibition of coxsackievirus B3 (CVB3) proliferation in cells nearly void of GSH. They made use of a drug, called BSO, that interferes with GSH biosynthesis [2] and they presented evidence suggesting that the block was in morphogenesis.

The mechanism of poliovirus morphogenesis is the least understood step in viral proliferation. Although the basic steps of PV morphogenesis have been established (Table 1), very little is known about the details of this process. We have recently studied steps in morphogenesis of PV1 and CAV20, which are C-cluster human enteroviruses, and have suggested that genome encapsidation occurs without the involvement of an RNA packaging signal; instead encapsidation specificity relies on the interaction between 2C^ATPase^ (in the replication complex) and capsid proteins VP3 and VP1 [21], [22].

In an effort to extend our understanding of the mechanism of enterovirus morphogenesis, we have undertaken a study of the role of GSH in enterovirus proliferation with the use of BSO, an inhibitor of GSH biosynthesis. Depletion of GSH with BSO is slow (48 hr) but highly efficient [3]. Notably, the nearly complete removal of GSH had no effect on CAV20 translation or processing and on genome replication. The same was found for PV1 infected GSH-depleted HeLa cells (data not shown). Yet the production of infectious PV1 and CAV20 virions was inhibited >100 fold.

We then searched for and found PV1 and CAV20 mutants (referred to as BSOr mutants) that replicated to high titers in GSH-depleted cells. Notably, the mutations in all BSOr variants mapped to capsid protein VP1 and VP3 (Figure 3A), and none to 2C^ATPase^. Moreover, nearly all mutations mapped to interfaces of protomers within the pentamers (Figure 4). Failure to score escape mutants in 2C^ATPase^ was at first disappointing but it suggested that the interaction between 2C^ATPase^ and VP3/VP1 is not affected by GSH. The PV type 1 Sabin, which is BSO-resistant, contains mutations in all of the capsid proteins but it is not known which one is responsible for the BSOr phenotype. Interestingly, the temperature sensitive phenotype of PV type 1 Sabin is due to a defect in the assembly of 14S particles and mature virions [30].

A key observation in this study was that the mature wt virus and capsid precursors of both PV1 and CAV20 were all pulled down by GSH-Sepharose beads (Figure 6). This indicated a direct interaction of the viral structural components and of the virion with GSH. GSH depletion with BSO eliminated those interactions (Figure 6). However, virus particles derived from BSOr variants, grown in GSH-depleted cells in the presence of BSO, were fully capable of interacting with GSH (Figure 6). All of these data strongly indicate that the interaction of GSH with capsid proteins is important for producing infectious virus particles.

At what stage does GSH play its role in C-cluster enterovirus morphogenesis? We have infected normal and GSH-depleted HeLa cells with PV1-wt and PV1-BSOr and analyzed progeny virus and packaging intermediates by sucrose gradients (Figure 7). Infection of untreated cells produced the well-known patterns of 5S, 14S, 75S and 150S peaks, a signature of normal morphogenesis intermediates. In BSO-treated cells, however, an adequate accumulation of 14S pentamer was reproducibly missing. Moreover, in the 150S region, some material sedimenting slightly faster than wt 150S virions accumulated. This material contained very little infectivity and it did not survive purification and analysis in CsCl gradients.

These data suggested that GSH has very important functions in morphogenesis by promoting the formation of stable capsid precursors and of the mature virus. In particular, GSH appears to be required for the accumulation of 14S pentamers. There are two possible explanations for the reduced amounts of 14S material visible on the gradient. First, without GSH the 14S pentamers are unstable, which, however, still associate with RNA and form non-infectious particles that sediment faster than virions. Alternatively, the unstable pentamers are depleted from the lysate by being rapidly incorporated into 75S empty capsids, which normally are the transient storage forms of pentamers that spontaneously assemble and disassemble [14], [17]–[20]. However, in the presence of the drug, the inability of 75S particles to bind GSH indicates some type of abnormality in their capsid structure or conformation that makes them unable to dissociate back to 14S.

Overall, the failure of these capsid precursors to bind to GSH, their mobility shift in sucrose gradients, and their instability in CsCl gradients, clearly indicate that GSH is required for maintaining their structure or conformation, particularly that of the 14S pentamers. Early studies on PV capsid assembly suggested the existence of an “activated” 14S particle whose formation was dependent on a “morphopoietic factor” present in virus-infected cell extracts and to a lesser extent in uninfected HeLa extracts [31], [32]. Whether or not this factor was GSH remains to be determined.

It is interesting to note, in the context of 14S particle assembly, that a lack of myristolylation of VP0 also leads to a reduction or block in the assembly of protomers to pentamers [33]–[35]. It was suggested that the myristate moieties, clustered near the contact area between the 5 protomers that form the 14S particle, are involved in stabilizing the pentamers. Whether there is any type of interaction between the myristate group of VP0/VP4 and GSH in stabilizing these precusors is not known.

The BSOr variants appear to require no or little GSH for capsid stability, an observation indicating that the capsid mutations restore the proper protomer/protomer interactions that are required for the formation of stable 14S pentamers and, subsequently, of mature virus. Nevertheless, the BSOr variants retain their ability to bind GSH as evidenced by pull-down experiments. There are two possible explanations of these findings. First, that the GSH binding site(s) on the structure of the capsid is completely different than the locations of the BSO resistant mutations. Therefore the ability to bind GSH is not related in anyway to a requirement (or a lack of requirement) of GSH for growth. The BSOr mutant might not need to bind GSH for growth because its 14S particle and capsid are already stable enough for normal morphogenesis. Second, compared with the capsid precursors of the wt virus, the BSOr capsid precursors might have a higher affinity for GSH enabling the BSOr mutants to grow in BSO treated cells, which contain only trace amounts of GSH.

Our previous genetic and biochemical studies have established that the 2C^ATPase^ protein, a viral polypeptide central in genome replication, interacts with capsid proteins, particularly VP3, and that this interaction is essential for morphogenesis [21], [22]. At the height of poliovirus proliferation, both poliovirus genome replication and morphogenesis occur on peri-nuclear membranes of infected cells. Immunofluorescence cell imaging experiments shown here clearly indicate that in GSH-depleted cells, virions are not formed at any site in the infected cell, and particularly not on peri-nuclear membranes. Normal replication is seen, however, in cells infected with PV1-BSOr variants regardless of whether the cells were GSH-depleted or not (Figure 8A). In contrast, capsid protein VP3 and polypeptide 2C^ATPase^ that co-localize in the peri-nuclear membranes during ordinary infection, are still found together in normal and GSH-depleted cells, irrespective of whether the cells were infected with PV1-wt or BSOr (Figure 8B). Therefore, whatever influence GSH depletion has on the structures of the capsid related particles this does not prevent normal association of 2C^ATPase^/replication complex with the VP3 polypeptide contained in these particles. This is consistent with the observation that capsid proteins obtained from BSO-treated cells can still associate with RNA to form 150S, albeit unable to produce mature virion.

Does the depletion of GSH induce an alternative, hostile condition (other than lack of GSH) in the host cell that would interfere with capsid assembly, such as turnover of host factors essential for morphogenesis? We cannot rigorously exclude such scenario. However, the inhibitory state of BSO-treated cells can be reversed by adding GSH to the cell culture 2 hr after infection and harvesting for progeny virus after a single growth cycle. Although replication cannot be restored to wt levels under the conditions of the experiment, synthesis of progeny to titers of 7 logs makes it unlikely that essential host factors were synthesized during the short time. Unexpectedly, DTT, a reducing agent normally not present in infected cells, was also able to partially reverse the effect of BSO-treatment. We assume that the effect of DTT is indirect, for example it might function by reducing GSSG, which accumulated in the cell during GSH starvation, to GSH. It should be noted that DTT does not reverse the effect of GSH-depletion during CVB3 infection of cells [3].

Heating of poliovirus particles causes extensive alterations to their physical and biological properties [36]–[38]. Heat-inactivated virus particles lose VP4 and are not able to adsorb to host cells thereby losing infectivity and their antigenic properties are changed [36]. Early studies on the stability of PV have shown that *in vitro* heat inactivation of the virus can be prevented by the addition of thiol compounds such as GSH or S7 (ethyl-2-methylthio-4-methyl-5-pyrimidine carboxylate) [10]. Our current experiments indicate that both GSH and GSSG are able to protect PV1-wt from heat-inactivation but DTT or β-ME cannot. These results suggest that the reducing ability of GSH is not important in preventing heat-inactivation but that direct binding increases heat stability. Compared to the wt virus, the BSOr variant is much more resistant to heat inactivation and exhibits less dependence on GSH for protection from heat inactivation. Therefore, it is likely that the BSOr variants are particles of increased physical stability formed by bypassing the inadequate GSH supply.

**Figure 10:**
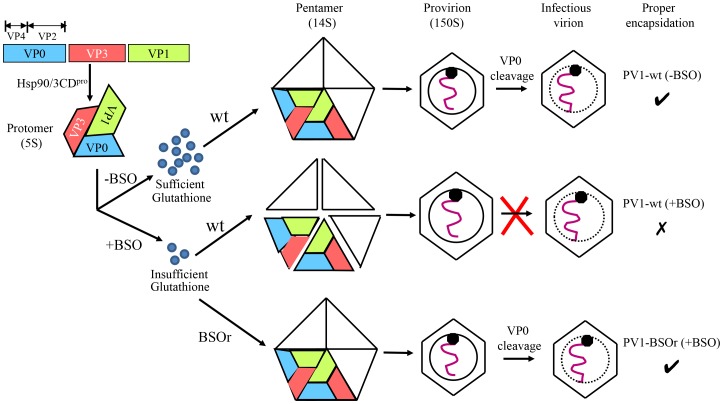
Model for the role of GSH in the morphogenesis of C-cluster enteroviruses. Capsid precursor P1, in a complex with Hsp90, is processed by 3CD^pro^ yielding mature viral proteins VP0, VP1 and VP3, which spontaneously form 5S protomer. Under normal growth conditions, with ample supply of GSH, 5 protomers assemble into a pentamer (14S). The pentamers then either form an empty capsid or condense around the progeny RNA and form a provirion. The mature virus is derived from the provirion after the cleavage of VP0 to VP2 and VP4. When GSH levels are low, due to the presence of BSO, the pentamer is unstable. However, these unstable 14S pentamers can associate with RNA and form provirion-like particles, which can't mature into an infectious virus. The capsid of the BSOr mutants is more stable than that of the wt virus and requires much lower levels of GSH for the assembly of the precursors and the mature virus.

Based on our results we propose the following model for the role of GSH in morphogenesis (Figure 10). Under normal growth conditions GSH binds to and stabilizes the 14S particles and enhances their assembly into the provirion/virion after association with RNA. In the presence of BSO the GSH concentration in the cell is too low for the stabilization of the pentamers, which may fall apart. In contrast, PV1-BSOr mutants, whose capsid proteins may have higher affinity to GSH, do not have difficulty assembling under low GSH concentrations and form more stable 14S pentamers.

## Materials and Methods

### Materials

L-buthionine-S,R-sulfoximine (BSO), Glutathione (GSH), oxidized Glutathione (GSSG) and DL-Dithiothreitol (DTT) were purchased from Sigma-Aldrich. ^35^S-Translabel (1000 C/mmol), containing labeled methionine and cysteine, and Actinomycin D were products of MP Biochemicals. Guanidine HCl, GSH Sepharose 4B, and Sepharose 4B, were purchased from Qiagen, BioWorld, and GE, respectively.

### Plasmids

pT7PV1(M) and pT7CAV20 contain the full-length infectious cDNA of PV1(M) and CAV20, respectively [39], [40]. PV(Sab) consisting of the cDNA of full-length infectious PV type 1 Sabin [24] was a gift of Dr. Akio Nomoto. The CAV20-FLuc plasmid was generated by replacing the P1 coding region of CAV20 with the firefly luciferase gene (FLuc). A 2A^pro^ cleavage site is inserted between coding sequence of FLuc and the P2 nonstructural polyprotein.

### Cells and viruses

HeLa H1 or HeLa R19 cells were maintained in Dubecco's modified Eagle's medium (DMEM) (Life Technology) supplemented with 8% bovine calf serum (Thermo Scientific), 100 units/ml of penicillin-streptomycin (Life Technology). Poliovirus type 1 (Mahoney) PV1-wt and coxsackievirus A20 (CAV20), two C-cluster enteroviruses, were derived from their respective cDNAs, pT7PVM and pT7CAV20. PV type 1 Sabin virus was derived from the PV(Sab) cDNA.

### Plaque assays

Plaque assays were performed in HeLa R19 cells using 0.6% tragacanth gum [41]. The plates were incubated for 48 hr at 37°C and were stained with 1% crystal violet.

### Isolation of PV1 and CAV20 BSO resistant mutants

BSO-starved HeLa cells were infected with PV1-wt at a moi (multiplicity of infection) of 0.01 and incubated in the presence of BSO for 24 hr at which time partial CPE was observed. The infected cells were lysed and an aliquot of the lysate was passaged on fresh BSO-treated cells. PV1-BSOr mutants evolved rapidly, already during the first passages on BSO-treated HeLa cells. The PV1-BSOr mutants were plaque purified and the RNAs were subjected to RT-PCR and sequence analysis. Thirteen independent plaques were sequenced in the P1 domain and found to contain three types of mutations (Fig. 3A). For the isolation of CAV20-BSOr mutants BSO-treated HeLa cells were infected with CAV20 at a moi of 1–2 and incubated in the presence of BSO. After 24 hr incubation the infected cells were lysed and an aliquot was used to infect fresh BSO-treated cells. CAV20-BSOr appeared after 3 passages on HeLa cells. The CAV20-BSOr mutants were plaque purified and the RNAs were subjected to RT-PCR and sequence analysis. The entire RNA genome of four independent plaques was sequenced and only one type of mutation was observed (Figure 3A).

### RT-PCR and sequencing analysis of viral RNA

Plaque purified viruses were used to infect HeLa cells and at 7 hr postinfection, total cytoplasmic RNAs were extracted with 1 ml Trizol reagent (Invitrogen). They were reverse transcribed and amplified into cDNA using the Titan one tube RT-PCR system (Roche). The cDNA products were sequenced using the Big Dye Terminator sequencing Kit (ABI).

### Growth curves of PV1-wt, PV1-BSOr, CAV20, CAV20-BSOr viruses

HeLa cells, untreated or pretreated with 0.4 mM BSO for 48 to 72 hr, were infected with PV1-wt or PV1-BSOr, CAV20, CAV20-BSOr or PV type 1 Sabin virus at a moi of 10. Incubation was continued in the presence of 0.4 mM BSO. Cultures were harvested at 0, 2, 4, 8 hr post-infection and the virus titers were determined by plaque assays. The Sabin1 culture was harvested at 8 hr post-infection.

### *In vitro* translation

*In vitro* translations were performed with HeLa cell-free extracts at 34°C, as described previously [41]. The viral proteins were analyzed by SDS-polyacrylamide gel electrophoresis (SDS-PAGE) containing 11% or 12.5% acrylamide.

### ^35^S-Labeling of CAV20 viral proteins *in vivo*

HeLa H1 cells were either untreated or pretreated with 0.4 mM BSO for 48 to 72 hr before infection with CAV20 at a moi of 10. Incubation was continued in the presence of 0.4 mM BSO. The viral proteins were labeled with ^35^S-Translabel (a mixture of methionine and cycteine) 4–6 hr post-infection and were analyzed by SDS-PAGE.

### Viral RNA synthesis

HeLa H1 cells were pretreated with BSO for 48 to 72 hr and transfected with the CAV20-Fluc replicon RNA in the presence of 0.4 mM BSO. Incubation was continued in the presence of 0.4 mM BSO. When indicated, 2 mM GnHCl (guanidine hydrochloride) was added at the time of transfection. RNA replication was measured at 9–12 hr post-transfection by measuring the luciferase signal (Promega) in an Optocomp I luminometer (MGM).

### Transencapsidation assay of CAV20-Fluc replicon RNA

Transcript RNA of the CAV20 replicon was transfected into BSO-pretreated HeLa H1 cells and incubated in the presence of 0.4 mM BSO. At 1 hr post-transfection, the cells were superinfected with either CAV20-wt or CAV20-BSOr at a moi of 0.5 and incubation was continued in the presence of 0.4 mM BSO. Cells were lysed at 6 hr postinfection by freezing and thawing and the lysates were used to re-infect fresh HeLa cells. Luciferase signal was determined at 6 hr post-infection.

### Sedimentation analysis of virus capsid intermediates by sucrose gradient fractionation

Briefly, untreated or BSO-pretreated HeLa cells in 15 cm dishes were infected with PV1-wt at a moi of 10. 50 µl Actinomycin D (1 mg/ml) was added 1 hr after infection to the cultures. At 4 hr postinfection, 200 µCi of ^35^S-Translabel was added and incubation was continued for 2 hr. The labeled cells were washed and harvested in TNM buffer (10 mM Tris pH 7.5, 10 mM NaCl, 1.5 mM MgCl_2_, 0.1% Tween). After freeze/thawing the supernatants were applied to a 6 to 25% or to a 15 to 30% sucrose gradient. The 6 to 25% gradients were centrifuged at 39,000 rpm for 16 hr in a SW41 rotor (Beckman) for the separation of the 5S/14S intermediates. The 15 to 30% sucrose gradients were centrifuged for 2.5 hr for the separation of 75S/150S intermediates. For each gradient, about 30 fractions (400 µl) were collected from the top and the radioactivity was quantified by liquid scintillation counting (Perkin Elmer).

### Purification of ^35^S-labeled virus by CsCl centrifugation

Eight 15 cm plates of HeLa R19 cells, each containing 4×10^7^ cells, were starved for methionine and cysteine in DME (-Met, -Cys). The cells were infected with PV1-wt at a moi of 50 and after one hour 50 µg/ml Actinomycin D was added to each plate. At 4 hr post infection 200 µCi of ^35^S-Translabel was added to each plate and the incubation was continued for 2 hr more. The cells were harvested, washed with DME, and lysed by freezing & thawing. The cell debris was removed by centrifugation, and to the supernatant, SDS and EDTA were added to a final concentration of 1% and 4 mM, respectively. The mixtures were centrifuged for 3.5 hr at 30,000 RPM (TY 50.2 Ti, Beckman) at 21°C. The pellet was resuspended in 0.4 ml of PBS and mixed with 2.2 g CsCl in 4.5 ml PBS. The mixture was centrifuged at 40,000 RPM for 18 hr in a Beckman SW50.1 rotor at 21°C. An identical sample, which was made from cells treated with BSO, was run in a parallel tube. The virus band isolated from the −BSO gradient was dialyzed against PBS.

### GSH-pull down assay

100 µl of Glutathione Sepharose 4B beads (GE Health Life Science) were washed 3 times with 500 µl of TNM buffer containing protease inhibitor cocktail tablets (Roche) before incubation with the protein samples. After overnight incubation with viral proteins at 4°C, the Glutathione Sepharose 4B beads were washed with 500 µl TNM buffer 6 times. The protein samples bound to the beads were analyzed by SDS-PAGE. In all GSH-pull down assays, Sepharose 4B beads were used as control.

### GSH-pull down assay of *in vivo* ^35^S labeled viral proteins

250 µl of *in vivo* ^35^S labeled virus infected cell lysate (about one forth of total lysate harvested from 35 mm dish) was mixed with 100 µl of GSH-sepharose 4B beads or Sepharose 4B Beads and subjected to a GSH-pull down assay.

### GSH-pull down assay of mature virus

CsCl purified virus 50 µl (1/15^th^ of the band) was incubated either with Sepharose B beads or GSH Sepharose beads and subjected to a GSH-pull down assay.

### GSH-pulldown assay of capsid intermediates

Aliquots of the 5S, 14S, 75S and 150S peaks, separated by sucrose gradient centrifugation, were analyzed by SDS PAGE. Approximately equal amounts of each peak, estimated by the quantity of label in the VP3 bands on the gel, were used for the GSH-pull down assays.

### Immunofluorescence cell imaging

HeLa cells, untreated or BSO treated, were infected with wt or BSOr virus at a moi of 10 and were incubated for 4 hr at 37°C, in the absence or presence of 0.4 mM BSO, respectively. The infected cells were probed for mature virus with A12 primary antibody, which binds specifically to mature virus, and Alexfluor 488-conjugated secondary antibody. A12, kindly provided to us by Nihal Altan-Bonnet, is a monoclonal antibody originally isolated and characterized by Chen et al. [42]. Capsid precursors were probed by polyclonal VP3 antibody. The localizations of 3AB and 2C^ATPase^, members of the replication complex, were determined in the same cell using 3AB and 2C^ATPase^ mouse monoclonal antibodies (isolated in our laboratory) and Alexfluor 555-conjugated secondary antibody. The scale is 5 µm.

### PV heat-inactivation assay

3×10^9^ PFU of PV1-wt or BSOr viruses were diluted in 200 µl PBS and were incubated at 48°C for 25 min in the absence or presence of GSH and other chemical reagents. Samples were cooled in ice and the virus titers were determined by plaque assay.
